# Novel small peptides derived from VEGF_125-136_: potential drugs for radioactive diagnosis and therapy in A549 tumor-bearing nude mice

**DOI:** 10.1038/s41598-017-04513-y

**Published:** 2017-06-27

**Authors:** Xiang Zhang, Shibin Feng, Jie Liu, Qianwei Li, Lei Zheng, Laiping Xie, Hongmin Li, Dingde Huang

**Affiliations:** Department of Nuclear Medicine, Southwest Hospital, Third Military Medical University, 30 Gaotanyan Street, Shapingba District, Chongqing, 400038 China

## Abstract

Vascular endothelial growth factor receptor (VEGFR) is a critical factor in tumor angiogenesis and has been considered a potential target for receptor-mediated radionuclide imaging and therapy. In this study, we identified two peptides (QKRKRKKSRKKH and RKRKRKKSRYIVLS) derived from VEGF_125-136_ that displayed high binding affinities to VEGFR and strong inhibition of A549 cell growth. ^99m^Tc- and ^188^Re-labeled peptides displayed high labeling efficiency and favorable stability in saline and human plasma. At the cellular level, the radiolabeled peptides could bind with A549 cells and be internalized via the VEGFR-1-mediated pathway. ^99m^Tc/^188^Re-labeled peptide was significantly accumulated at xenograft tumors, as observed with single-photon emission computed tomography (SPECT) planar imaging. Moreover, ^188^Re-labeled peptides significantly inhibited tumor growth, prolonged the survival time of the tumor-bearing nude mice and resulted in much more necrotic regions and apoptotic cells in the A549 xenograft tumors. These results demonstrated that these two peptides as candidate drugs for radionuclide imaging and tumor therapy.

## Introduction

Molecular imaging enables the detection and visualization of cancer-related biomarkers in tumors^1^. This technique can facilitate the early and accurate detection of cancer, supporting the use of molecular signatures to tailor effective and personalized therapies for improved patient survival^2^. Molecular probes or tumor-targeted drugs could be labeled with different types of imaging agents for tumor imaging and therapy. Small molecular tracers labeled with radionuclide can be detected non-invasively by positron emission tomography (PET) or single photon emission computed tomography (SPECT) with high sensitivity. In addition, local/regional radiotherapy could be achieved using therapeutic radionuclide-labeled tumor-targeted drugs or peptides^3, 4^.

Angiogenesis is essential in the process of primary tumor growth, proliferation and metastasis^5, 6^. One of the best characterized and vital groups of pro-angiogenic protein factors includes the members of the vascular endothelial growth factor (VEGF) family, consisting of VEGF-(A-D) and placenta growth factor (PIGF). Of these, VEGF-A is principally responsible for vessel formation in adult tissues^7^. VEGF binds to VEGF receptors (VEGFRs) with three isoforms: VEGFR-[1–3]^8^. VEGF binds with higher affinity to VEGFR-1; however, its primary effects on angiogenesis are mediated by VEGFR-2, the primary receptor involved in endothelial cell proliferation and migration; VEGF binding to VEGFR-2 stimulates downstream signal transduction, leading to endothelial proliferation, differentiation, permeability, migration and the generation of new blood vessels^9, 10^. Compared with normal cell types, VEGFRs are more highly expressed in tumor cells and in newly formed vascular endothelial cells^11, 12^, which suggests that VEGF analogues are potential targeting drugs for receptor-mediated imaging^13, 14^.

As the VEGF/VEGFR pathway is mainly responsible for tumor angiogenesis, VEGFRs are the most important factors contributing to tumor growth, angiogenesis and lymphangiogenesis. Targeting tumor angiogenesis has been approached through monoclonal antibodies that block VEGF-VEGFR binding^15–17^, and several drugs that block the VEGF/VEGFR pathway, such as bevacizumab and ramucirumab, have been approved by the FDA for treatment of common solid tumors^18–21^. However, major limitations of angiogenesis inhibitors have been observed by continued clinical investigations, such as resistance, enhancing tumor hypoxia and reducing the delivery of chemotherapeutic agents, which might be the main reasons for poor improvement after the clinical administration of angiogenesis inhibitors^22^.

Peptide-based radiopharmaceuticals for tumor imaging and therapy have been rapidly developed because of their many unique advantages, such as high receptor binding affinity, high tumor penetration, favorable pharmacokinetics (rapid removal from the blood pool) and few side effects^23, 24^. VEGF_125-136_ (QKRKRKKSRYKS), a 12 amino acid peptide encoded by exon 6 of VEGF-A, was identified as an effective inhibitor of VEGF binding to VEGFR *in vitro*, early biological effects of VEGF and VEGF-dependent angiogenesis^25^. Furthermore, *in vivo* evaluation indicated that the peptide was a highly effective anti-angiogenesis agent that competed with VEGF and suppressed ischemic retinal neovascularization as effectively as soluble VEGFR-1^26^. In our previous study, ^188^Re-labeled VEGF_125-136_ exhibited good tumor targeting effect *in vivo* and could be used as a highly specific agent for tumor radionuclide imaging and therapy^27^. Furthermore, with the aim to improve target capability for tumor theranostics, we optimized VEGF_125-136_ *via* bioinformatics prediction and *in vitro* cell experimentation and obtained two peptides (QKRKRKKSRKKH and RKRKRKKSRYIVLS) with higher affinity to VRGFR than VEGF_125-136_^28^. In the present study, these two peptides were labeled with ^99m^Tc and ^188^Re, and *in vitro* and *in vivo* experiments were performed to evaluate their application for tumor imaging and therapeutic effect on A549 tumor-bearing nude mice; the underlying mechanism was also preliminarily explored.

## Results

### Screening for optimal peptides

**Figure 1:**
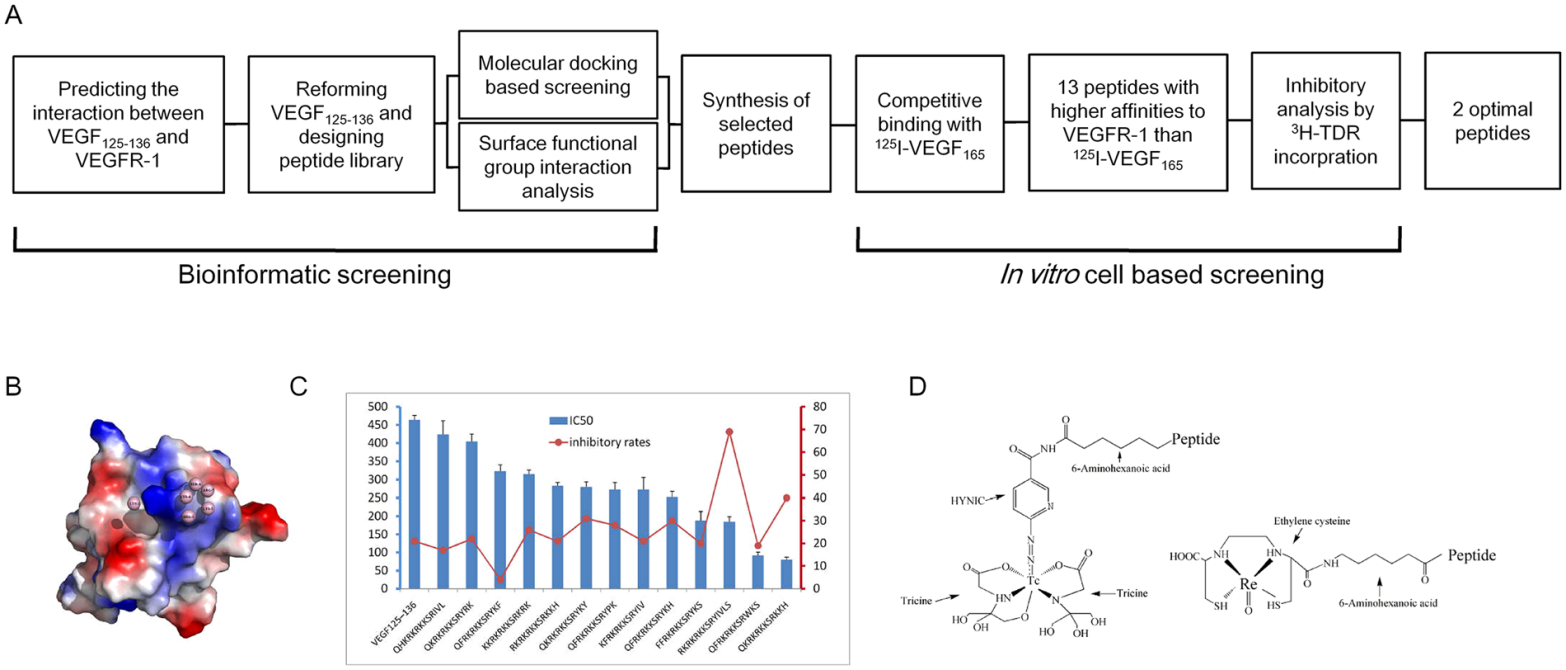
Screening of optimal peptides. (**A**) Flow chart for peptide screening via bioinformatics and cell-based assays. (**B**) Simulated image of VEGFR-1 interacting with the binding core of VEGF_125-136_. (**C**) *In vitro* competitive binding of the 13 selected peptides with ^125^I-VEGF_165_ and the ^3^H-TDR incorporation assays. (**D**) Structures of the HYNIC- and EC-conjugated peptides.

Thirteen peptides exhibited lower IC_50_ values than VEGF_125–136_ (464 nmol/L). The IC_50_ values of QKRKRKKSRKKH and RKRKRKKSRYIVLS were significantly lower than that of VEGF_125–136_, at 80 nmol/L and 185 nmol/L, respectively. These values correspond to 6- and 2.5-fold increases in affinity for QKRKRKKSRKKH and RKRKRKKSRYIVLS, respectively. The inhibitory rates of the 13 peptides are shown in Fig. 1C, QKRKRKKSRKKH and RKRKRKKSRYIVLS displayed low IC_50_ values (79.75 ± 7.03 and 185.24 ± 14.18 nmol/L, respectively) as well as high inhibitory rates (40% and 70%, respectively), which indicates that these compounds were the optimal peptides after serial screening. Blast searches of the NCBI database revealed that both peptides were novel. Therefore, QKRKRKKSRKKH and RKRKRKKSRYIVLS were chosen for further evaluation.

### Radiochemical purity and *in vitro* stability of ^99m^Tc/^188^Re-labeled peptide

**Figure 2:**
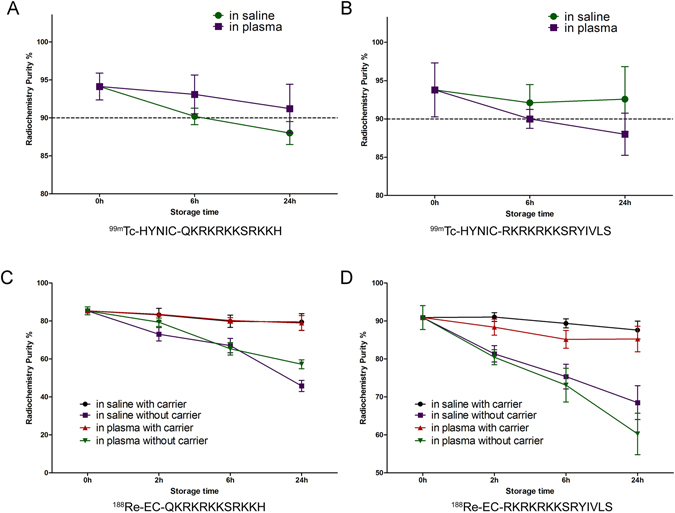
Stabilities of the ^99m^Tc-labeled and ^188^Re-labeled peptides. The stabilities of the ^99m^Tc-labeled (**A**,**B**) and ^188^Re-labeled peptides (**C**,**D**) were evaluated in saline and human plasma. The ^99m^Tc-labeled peptides maintained high stability in both solutions for 24 h. Ammonium perrhenate was used as a carrier, which increased the stability of the ^188^Re-labeled peptides.

The labeling rates of ^99m^Tc/^188^Re-peptides were detected by paper chromatography using ammonia-ethanol-water (1:2:5) and acetone as developing agents (Fig. S2). The radiochemical purities of the ^99m^Tc/^188^Re -peptides were around 90% (Table 1) and further evaluated by HPLC (Figs S3 and S4). The specific activities of ^99m^Tc-peptide and ^188^Re-peptide were as high as 16 TBq/mmol and 8 TBq/mmol, respectively. The radiochemical purity (RCP) of ^99m^Tc-peptides changed little following incubation with saline or human plasma for 24 h (Fig. 2A,B). The RCP values of ^188^Re-peptides in normal saline and human serum following incubation for 24 h were 79.43 ± 4.45% vs. 78.95 ± 3.88% and 87.6 ± 2.35% vs. 85.25 ± 3.36%, respectively (Fig. 2C,D). Of note, the stability of the ^188^Re-labeled peptides was significantly improved by the addition of ammonium perrhenate (carrier) after labeling.

**Table 1:** Labeling efficiency of peptide.

	^99m^Tc radiolabeling rate (%)	^188^Re radiolabeling rate (%)
QKRKRKKSRKKH	94.13 ± 1.77	85.34 ± 2.05
RKRKRKKSRYIVLS	93.79 ± 3.53	90.90 ± 3.45

### *In vitro* receptor competitive binding assays

**Figure 3:**
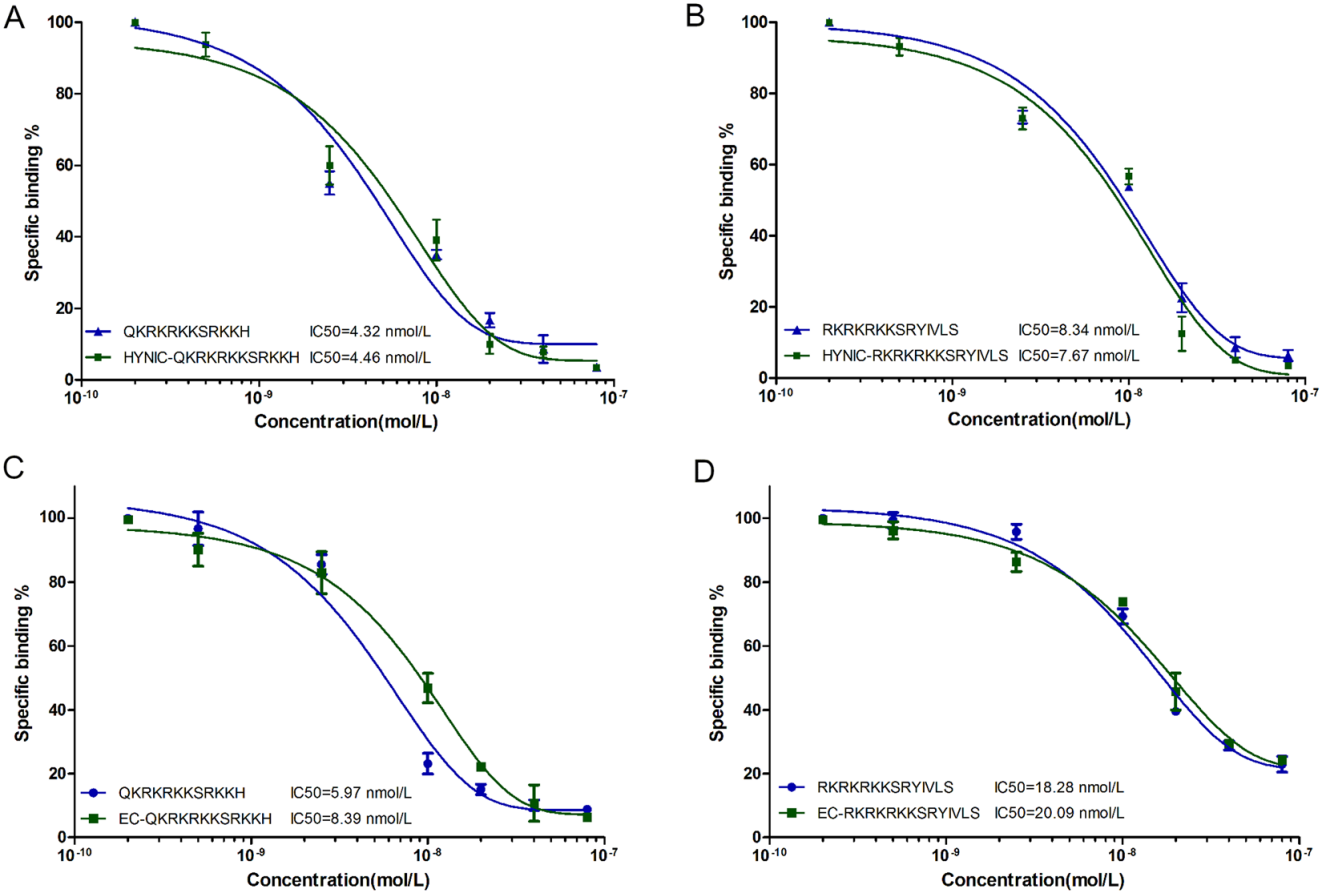
*In vitro* competitive receptor binding assays. The ^99m^Tc-labeled (**A**,**B**) and ^188^Re-labeled peptides (**C**,**D**) were incubated with the corresponding non-labeled peptides. IC50s of the radiolabeled peptides were calculated after competed by corresponding peptides and HYNIC/EC conjugated peptides.

Receptor competitive binding assays were performed to verify the binding affinities of the radiolabeled peptides. As shown in Fig. 3, both peptides potently inhibited the specific binding of the corresponding radiolabeled peptides to A549 cells in a concentration-dependent manner, indicating that the binding affinity of peptide to A549 cell was maintained after radionuclide labeling. Furthermore, there were no significant difference of IC50 between the non-conjugated peptides and the HYNIC/EC-conjugated peptides (Fig. 3), which indicated that chelating agents (HYNIC and EC) scarcely affect the binding abilities of the two peptides.

### Saturation binding, internalization assays and co-immunoprecipitation

Immunofluorescence staining (Fig. S5A) showed that a stronger fluorescent signal was detected in the transfected group than in the control group, indicating that VEGFR-1 was successfully transfected into A549 cells, and the transfection efficiency was as high as the MOI level of 50. RT-PCR (Fig. S5B) and western blot analysis (Fig. S5C) showed that VEGFR-1 expression values at the gene and protein levels were both significantly increased after transfection with Lenti-VEGFR-1.

**Figure 4:**
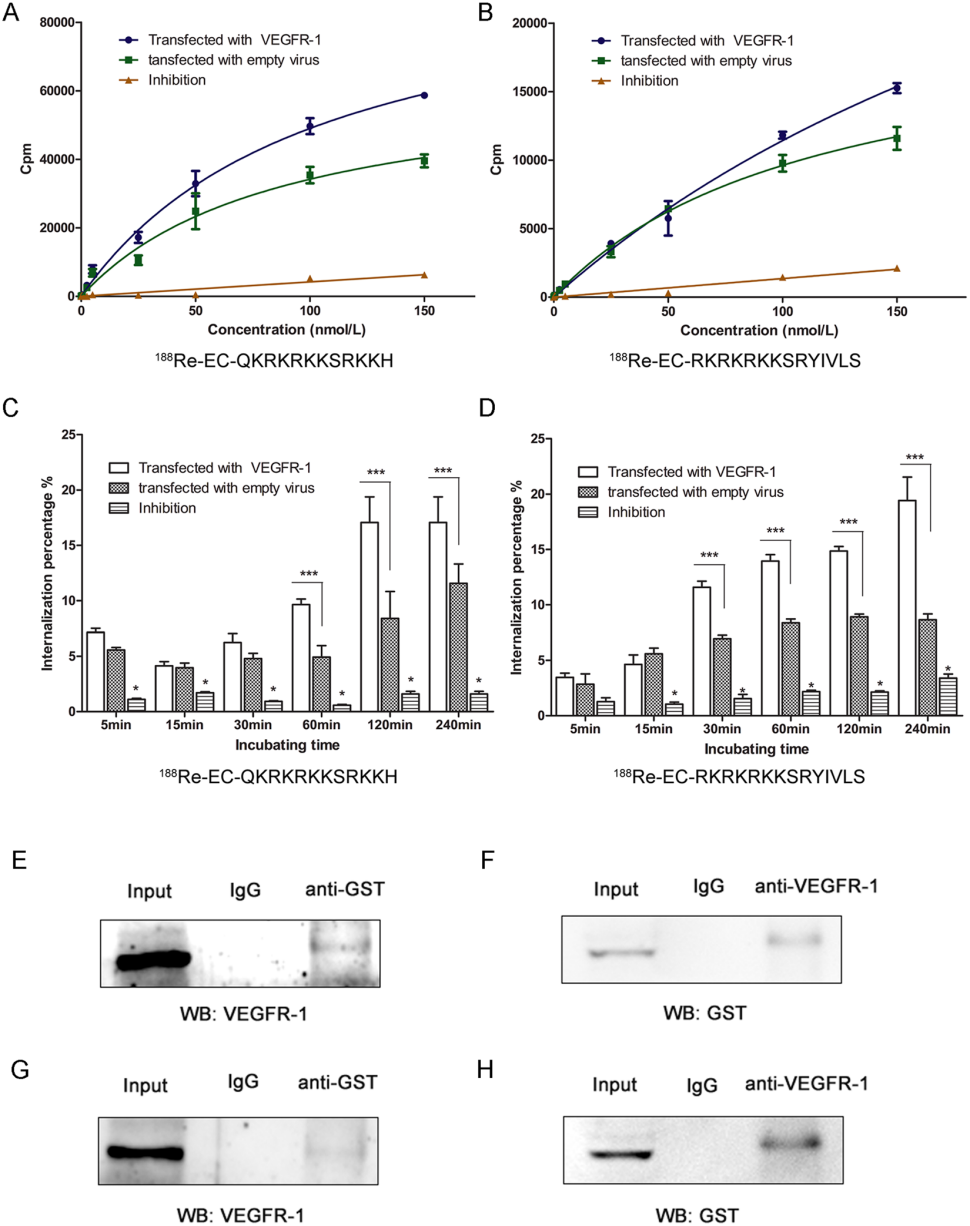
Binding assay between peptide and VEGFR-1. Receptor saturation binding assays (**A**,**B**) and internalization assays (**C**,**D**) of the ^188^Re-labeled peptides. VEGFR-1-overexpressing A549 cells displayed more abundant binding of the radiolabeled peptides and a higher internalization rate compared with the control cells. The inhibited group was compared with the other two groups at each time point. * *P* < 0.01; *** *P* < 0.001. (**E**,**F**) Co-immunoprecipitation of GST-QKRKRKKSRKKH and VEGFR-1; (**G**,**H**): Co-immunoprecipitation of GST-RKRKRKKSRYIVLS and VEGFR-1.

Experimental results revealed that the radioactivity of the A549 cells increased as the concentration of ^188^Re-labeled peptides increased and gradually became saturated. Of note, the saturation curve of the VEGFR-1-overexpressed A549 cell group plateaued later and higher than that in the control groups, indicating the binding specificity of the peptides to VEGFR-1 (Fig. 4A,B). Peptide internalization by A549 cells was also evaluated. The fraction of radiolabeled peptides that was internalized by A549 cell was significantly higher in the VEGFR-1-overexpressed cells than in the control group (Fig. 4C,D). The addition of excess non-labeled peptide significantly decreased radioactivity in each experiment.

Co-IP to test the combination between GST-QKRKRKKSRKKH and VEGFR-1: anti-GST antibody was used to immunoprecipitated the total protein, then Western blot showed that the precipitated protein complexes contained VEGFR-1 (Fig. 4E), then reversed Co-IP: anti-VEGFR-1 antibody was used to immunoprecipitated the total protein, Western blot showed that the precipitated protein complexes contained GST-peptide (Fig. 4F). So the result showed that GST-QKRKRKKSRKKH could bind to VEGFR-1.

Co-IP to test the combination between GST- RKRKRKKSRYIVLS and VEGFR-1: in a similar way, GST-RKRKRKKSRYIVLS could combined to VEGFR-1 (Fig. 4G,H), indicating that these two peptides could bind to VEGFR-1 and subsequently exert its biological effect on tumor cells.

Therefore, we concluded that the peptides could specifically bind to VEGFR-1 and be internalized by A549 cells *via* VEGFR-1-mediated pathway.

### SPECT planar imaging

**Figure 5:**
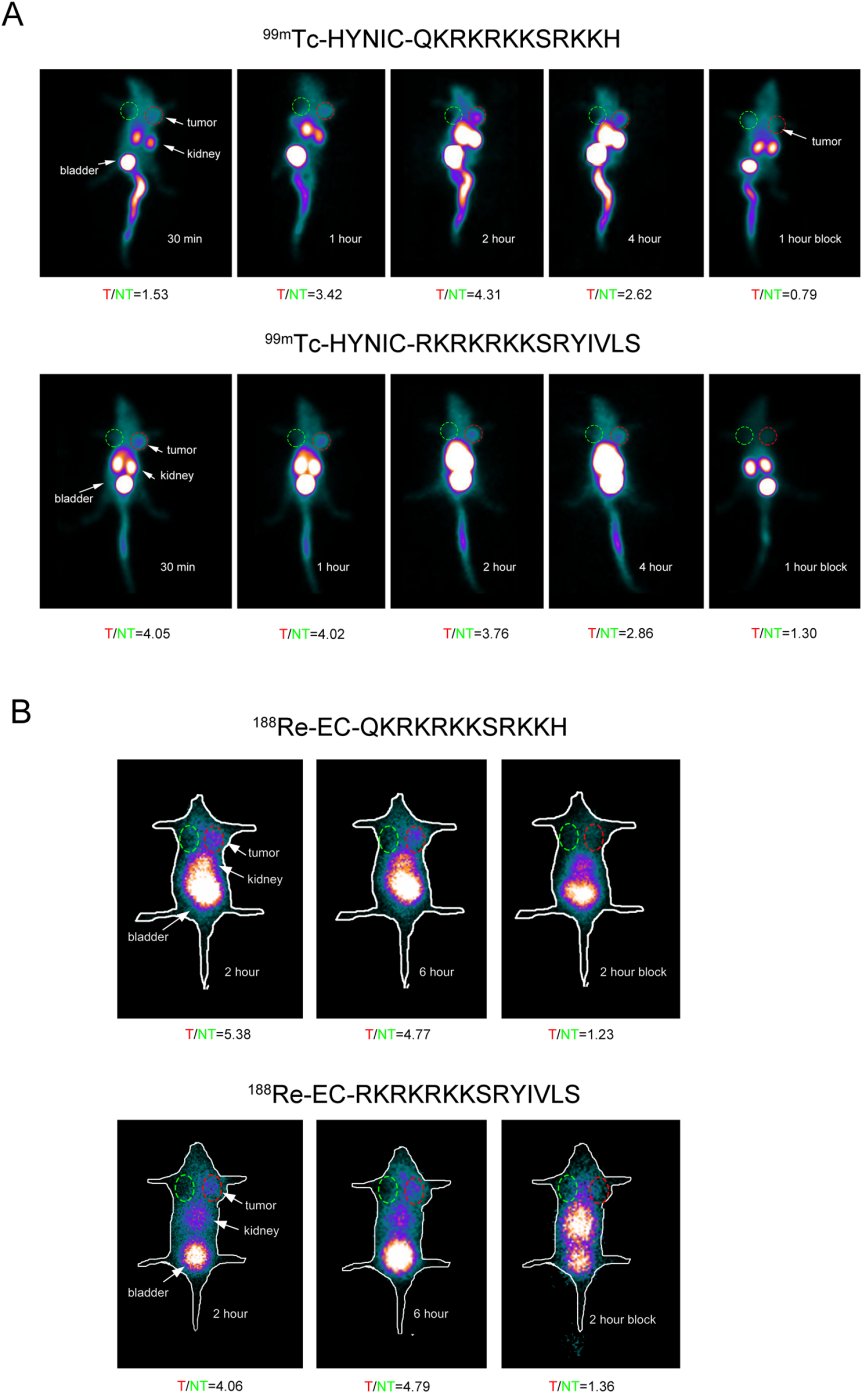
SPECT planar imaging of A549 tumor-bearing nude mice following the injection of ^99m^Tc/^188^Re-labeled peptides. Images were acquired at 0.5, 1, 2, 3, and 4 h following injection of the ^99m^Tc-HYNIC-peptides or at 2 and 6 h following the injection of the ^188^Re -EC-peptides. The tumor sites were clearly detected following injection (indicated by arrows on the image). Competitive binding was assessed as the absence of detection at 1 or 2 h following injection. Tumor/non-tumor (T/NT) ratio was showed below each image.

SPECT imaging was performed to examine the tumor target effect of ^99m^Tc/^188^Re-labeled peptides in A549 tumor-bearing mice (Fig. 5). ^99m^Tc-labeled peptides were accumulated at the tumor site as early as 30 min following *i*.*v*. injection, and the tumor area remained detectable at 4 hour. The tumor/non-tumor (T/NT) ratios were ranged from 1.53 to 4.31 for ^99m^Tc-peptides imaging and from 4.06 to 5.38 for ^188^Re-imaging at different time point. *In vivo* competitive binding considerably reduced the radioactivity and T/NT ratio in tumor-bearing nude mice, indicating that the binding of the peptides to tumor tissue was specifically mediated by the peptides. In addition, a high concentration of radioactivity was shown in the kidneys and in the bladder, suggesting that radionuclide-labeled peptides were excreted *via* the urinary system. No radioactivity was detected in the lung, brain or thyroid tissues. The results for ^188^Re-peptide imaging were consistent with the above-mentioned ^99m^Tc-peptide imaging.

### *In vivo* biodistribution of the radiolabeled peptides in tumor-bearing nude mice

**Figure 6:**
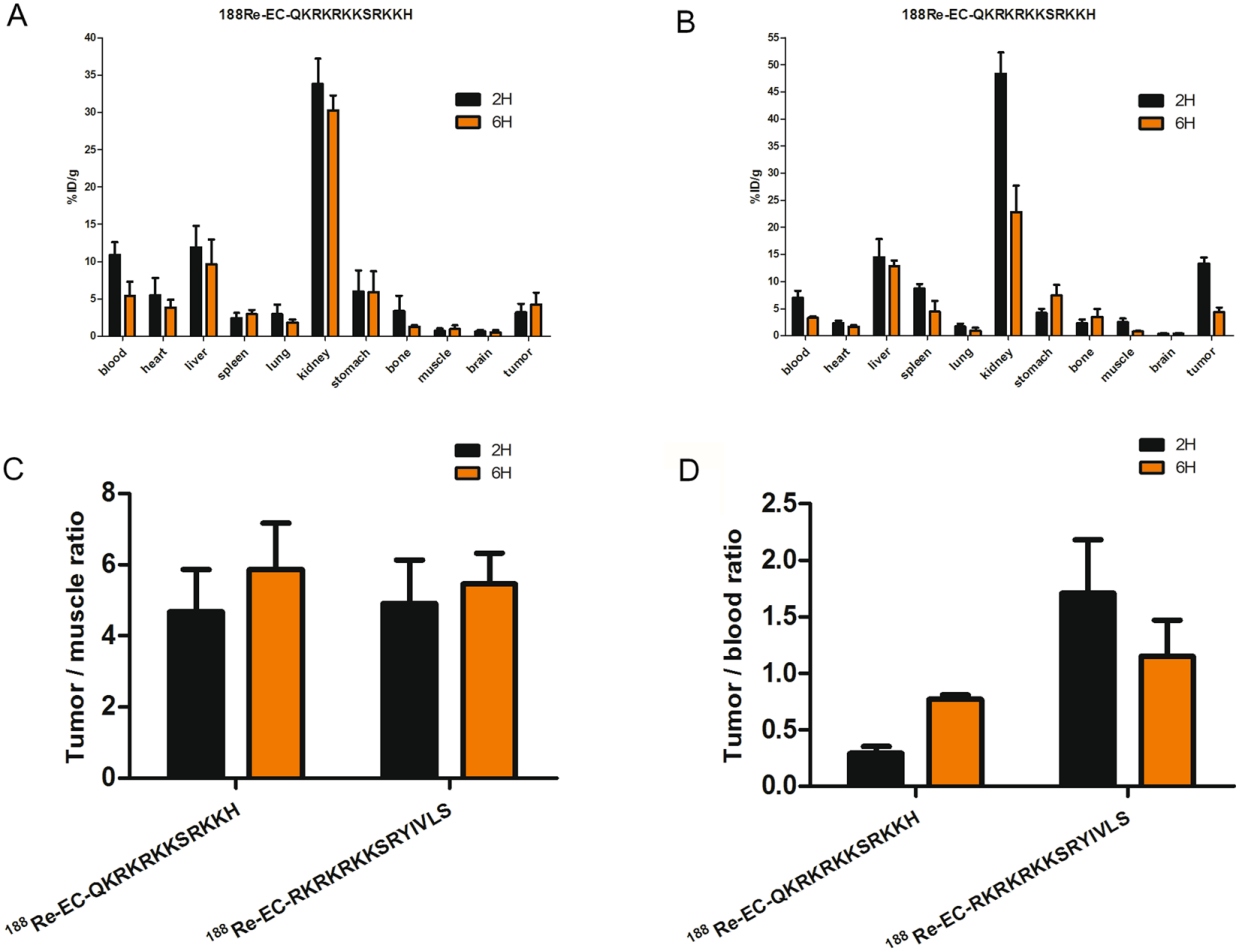
Biodistribution of ^188^Re-labeled peptide in A549 tumours. A549 tumor bearing mice received 7.4 MBq ^188^Re-labeled peptide (n = 12) intravenously. *Ex vivo* biodistribution at 2 and 6 h after injection of ^188^Re-labeled peptide are shown in figure (**A**,**B**). Tumor/muscle ratio (**C**) and Tumor/blood ratio (**D**) were calculated.

The biodistribution of the ^188^Re-labeled peptides in A549 tumor-bearing nude mice was evaluated at 2 h and 6 h following intravenous injection (Fig. 6A,B, Table 2). The tumor-to-muscle ratio (T/M ratio) of the 2 peptides reached 4.67 ± 1.2 and 4.91 ± 1.23 at 2 h, respectively (Fig. 6C). Additionally, the ^188^Re-labeled peptides were primarily distributed in the kidney, indicating that the radionuclide-labeled peptides were excreted via the urinary system. Meanwhile, high radioactivity levels were detected in blood, indicated a prolonged circulation time of ^188^Re-EC-peptides.

**Table 2:** Biodistribution data of ^188^Re-labeled peptides following intravenous injection into A549 tumor-bearing nude mice.

Organs	Uptake (%ID/g)				Tumor-organ ratio			
	QKRKRKKSRKKH		RKRKRKKSRYIVLS		QKRKRKKSRKKH		RKRKRKKSRYIVLS	
	2 h	6 h	2 h	6 h	2 h	6 h	2 h	6 h
blood	10.9 ± 1.72	10.9 ± 1.72	6.97 ± 1.36	3.26 ± 0.33	0.29	0.78	1.91	1.33
heart	5.5 ± 2.35	5.5 ± 2.35	2.31 ± 0.44	1.61 ± 0.3	0.58	1.09	5.76	2.70
liver	11.88 ± 2.92	11.88 ± 2.92	14.46 ± 3.38	12.85 ± 1.05	0.27	0.44	0.92	0.34
spleen	2.41 ± 0.7	2.41 ± 0.7	8.73 ± 0.8	4.41 ± 2	1.33	1.43	1.52	0.99
lung	2.93 ± 1.31	2.93 ± 1.31	1.69 ± 0.51	0.91 ± 0.57	1.09	2.32	7.88	4.78
kidney	33.79 ± 3.39	33.79 ± 3.39	48.33 ± 3.89	22.81 ± 4.84	0.09	0.14	0.28	0.19
stomach	6.02 ± 2.83	6.02 ± 2.83	4.18 ± 0.8	7.44 ± 1.94	0.53	0.71	3.18	0.58
bone	3.36 ± 2.09	3.36 ± 2.09	2.25 ± 0.75	3.45 ± 1.5	0.95	3.32	5.92	1.26
muscle	0.71 ± 0.34	0.71 ± 0.34	2.49 ± 0.7	0.79 ± 0.1	4.51	4.35	5.35	5.51
brain	0.61 ± 0.22	0.61 ± 0.22	0.34 ± 0.13	0.38 ± 0.15	5.25	7.96	39.15	11.45
tumor	3.2 ± 1.16	3.2 ± 1.16	13.31 ± 1.17	4.35 ± 0.85	—	—	—	—

### Therapeutic evaluation of the ^188^Re-peptides on A549 tumors

**Figure 7:**
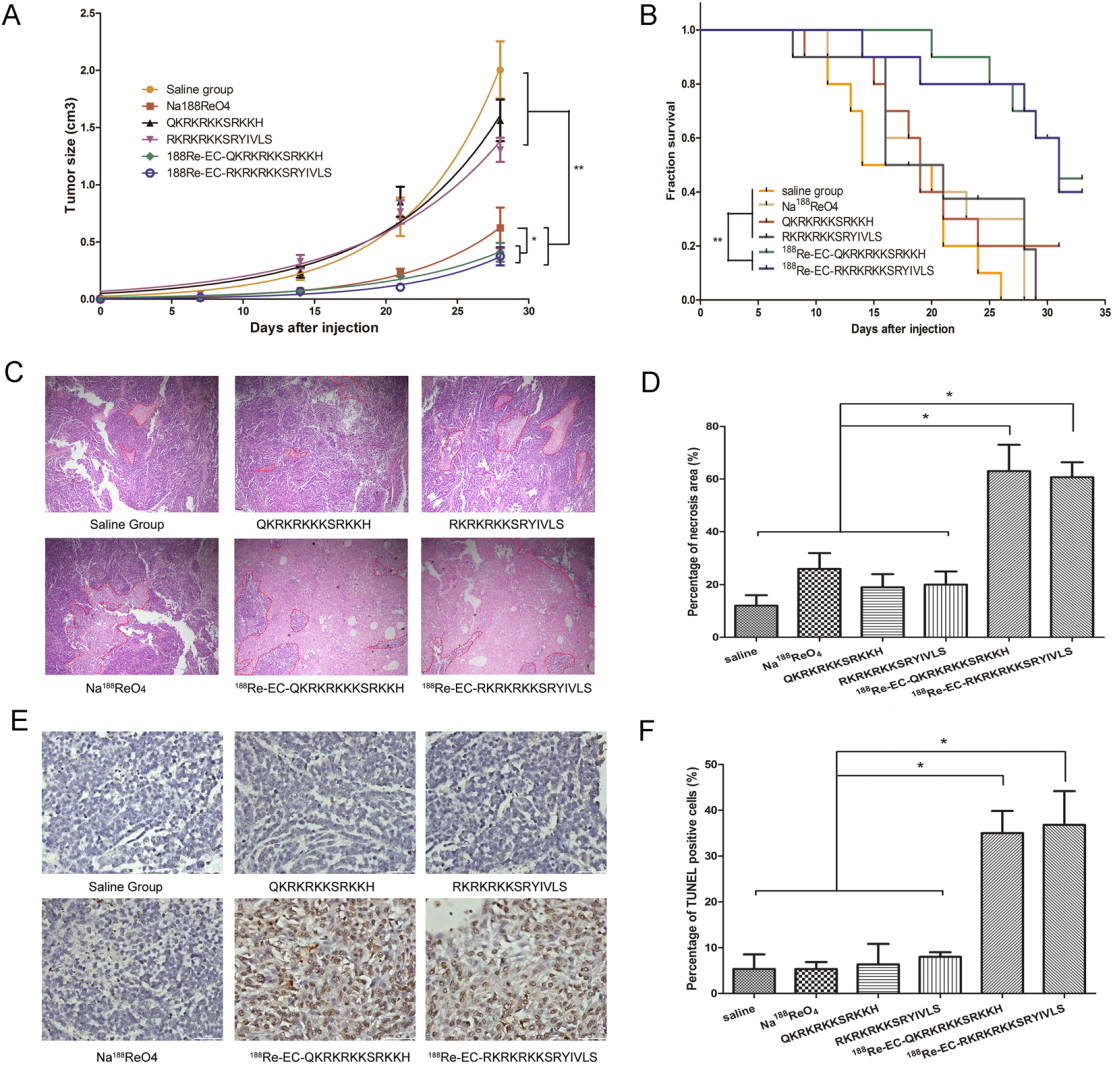
Therapeutic efficacy of the ^188^Re-peptides with respect to A549 xenograft tumor growth. (**A**) Tumor growth curves in each group of mice following treatment. The tumor volume was clearly reduced in the ^188^Re-labeled peptide-treated and ^188^Re-perrhenate-treated groups compared with the other groups post-treatment. (**B**) Survival curves of A549 tumor-bearing mice following the indicated treatments. (**C**) HE staining of A549 tumors from various treatment groups (×40). (**D**) Quantitative analysis was performed by estimating the proportion of the necrotic area. (**E**) TUNEL staining in non-necrotic zones (×400). (**F**) Quantitative analysis was performed by calculating the proportion of TUNEL-positive cells. *P < 0.05, **P < 0.01.

Compared with the saline group, in the ^188^Re-labeled peptide-injected groups, A549 tumor growth was significantly delayed (*P* < 0.05). However, there was no significant difference in tumor volume growth between the ^188^ReO_4_^−^ treated and ^188^Re-labeled peptide groups (Fig. 7A). The results of the survival analysis revealed significant differences between the ^188^Re-labeled peptide-treated and control groups (Fig. 7B), which indicated that the ^188^Re-labeled peptide displayed less toxicity than ^188^ReO_4_^−^ and a stronger therapeutic effect than the non-labeled peptides. HE staining of the tumor tissues revealed that there were more necrotic regions in the ^188^Re-labeled peptide-treated groups than in the control groups (Fig. 7C). TUNEL staining showed that higher level of apoptosis in the non-necrotic tumor areas of the ^188^Re-labeled peptide-treated groups than in other groups (Fig. 7D).

## Discussion

The key point of tumor-targeted radionuclide imaging and therapy is to utilize the targeting property of the molecule probe or drug to specifically deliver radionuclide to tumor sites, taking advantage of radioactive rays to detect or kill tumor cells. Ideal tumor-targeting drugs should display rapid clearance from plasma, high affinity to tumor cells and a long retention time at tumor sites. Thus, peptide- and receptor-mediated tumor imaging and therapy has recently become a hot topic of research^29, 30^. The first and most important step for the development of tumor-targeted peptides is the selection of the appropriate receptor, which is indicated by a high tumor/non-tumor ratio.

VEGFR is a critical factor in tumor angiogenesis and proliferation. VEGFR has been expressed at a high level in many types of malignant tumors, including lung cancer, breast cancer and neuroglioma^31, 32^. Many of these malignancies have a poor prognosis with advanced disease^33, 34^. Current treatments are suboptimal; therefore, there is heightened interest in the development of novel therapeutic strategies^35^. Thus, the overexpression/ectopic expression of VEGFR may represent a useful approach.

Peptides for receptor-mediated imaging and therapy should display at least two characteristics: high binding affinity and antagonism to the tumor. VEGF/VEGFR pathway is mainly responsible for tumor angiogenesis and growth. Different from VEGF, VEGF_125-136_ competes with wild type VEGF for VEGFR binding and subsequently blocks the VEGF/VEGFR signaling pathway, exhibiting its anti-angiogenesis effect. In the early stage of our study, we radiolabeled VEGF_125-136_ and demonstrated its tumor-targeting ability. However, the tumor/non-tumor ratio of this peptide was low and its retention time at tumor sites was short, which limited its applicability. Therefore, the optimization of this peptide was required. Compared with VEGF_125-136_, the polypeptides QKRKRKKSRKKH and RKRKRKKSRYIVLS exhibited 6- and 2.5-fold higher VEGFR binding affinity, respectively. Furthermore, these two peptides displayed a stronger inhibitory effect on tumor proliferation. After the optimization procedure, two polypeptides, QKRKRKKSRKKH and RKRKRKKSRYIVLS, were selected for further research.

Varieties of tumor cells express a high level of VEGFR-1, which contributes to their immortalization^36^. As previously described, the bioinformatics screening was based on the interaction of the peptides with VEGFR-1. To verify the accuracy of the bioinformatics prediction, a VEGFR-1 overexpression model was successfully constructed *via* transfection of a lentiviral vector. Receptor saturation, internalization assays and CO-IP were then performed. Compared with the control group, the radiolabeled peptides accumulated more strongly in the VEGFR-1 overexpression group and could permeate the cell membrane *via* VEGFR-1-mediated internalization. Furthermore, Co-IP was used to verify the specific binding between peptide and VEGFR-1; the results indicate that VEGFR-1 is a receptor of these two peptides.

A pair of radionuclides (^99m^Tc and ^188^Re) was used for radiolabeling. As technetium and rhenium belong to the same family of elements, they display somewhat similar physical and chemical properties. ^99m^Tc emits readily detectable 140 keV γ-rays, and its half-life is 6 h, which is well suited for SPECT imaging. In comparison, ^188^Re primarily emits high-energy β-rays that are suitable for radiotherapy *in vivo*. However, its inactive physical property leads to its stringent conditions for radiolabeling. Here, we chose ethylene cysteine as a chelating agent for peptide radiolabeling and screened for its optimal labeling conditions. The radiolabeling rates were as high as approximately 90%, but the RCP decreased to 70% after 24 h. This result was most likely caused by the reoxidation of ^188^Re. Nevertheless, the addition of ammonium perrhenate after the reaction increased the stabilities of these peptides in saline and plasma. This mechanism merits further investigation^37, 38^.

^99m^Tc/^188^Re-labeled peptides maintained high binding affinities to A549 cells, and the effects of conjugating HYNIC or EC, respectively, were evaluated simultaneously. The results suggested that the conjugation of a chelating agent or radionuclide did not affect the binding capacity of the peptides.

Following intravenous injection, the *in vivo* tumor targeting was assessed *via* SPECT, which has been widely used for clinical tumor detection. The tumor sites were clearly shown via planar imaging as early as 30 min following the injection of the radiolabeled peptide, and they remained detectable for approximately 6 h. Nevertheless, there was little difference in the tumor-muscle ratios between these two peptides. No clear radioactivity was detected in the thyroid tissue, which implied the chelating stabilities of the radiolabeled peptides. The high tumor uptake rates of these two ^188^Re-labeled peptides indicated their potential therapeutic effects *in vivo*. As expected, the ^188^Re-labeled peptides inhibited tumor growth as a result of the selective delivery of radionuclide to the tumor sites, which also significantly prolonged the survival duration of the tumor-bearing nude mice. Because of the cytotoxicity of ^188^Re, a large amount of necrosis and apoptosis was identified in the tumor tissues of the treated groups. It is important to note that the injection of an isodose of Na^188^ReO_4_ inhibited the tumor volume growth but did not improve the survival duration of the nude mice, and there was no significant difference in necrosis or apoptosis in the tumors.

The limitation of these radionuclide labeled peptides is their high uptake in blood, it revealed a prolonged circulation time of ^188^Re-EC-peptides. High radioactivity also observed in liver, kidney at 6 hour, it might affect the quality of abdominal imaging. Furthermore, high uptake in these organs might cause radiological toxicity after therapeutically injecting Re-188 labeled peptides. It is still necessary to further modify and purify these peptides to decrease the uptake in normal organs and increase the accumulation in tumor site. PEGylating^39^ peptides and modifying the peptides with hydrophilic amino acid might be possible ways to prolong tumor retention and improve the pharmacokinetic and pharmacodynamics properties.

## Conclusions

With bioinformatics prediction and *in vitro* experimentation, we screened two peptides with higher affinity to VEGFR-1 and stronger inhibitory effects on A549 cell growth. ^99m^Tc/^188^Re-labeled peptides with high RCP displayed a high accumulation in tumor tissue in tumor-bearing nude mice; furthermore, ^188^Re-labeled peptide treatment exhibited favorable therapeutic effects on tumors. This study provides a strong experimental foundation for further research on VEGFR targets for tumor imaging and therapy.

### Materials and Methods

#### Materials, cells and animal models

Na^99m^TcO_4_ was obtained from a commercial ^99^Mo/^99m^Tc generator (Beijing Atom High Tech Co., Ltd., Beijing, China), and Na^188^ReO_4_ was obtained using an ^188^W/^188^Re generator (ITG, Germany). Tris (3-sulfophenyl) phosphine trisodium salt (TPPTS) was purchased from J&K Chemical LTD. Tricine was obtained from Tokyo Chemical Industry Co., LTD. Stannous chloride and ammonium perrhenate were purchased from Sigma-Aldrich. SPECT (Millennium MPR, GE, USA); γ-counter (GAMMA-10, Shin Jin Medics Inc., Korea); HPLC (Agilent, USA); and the liquid scintillation counter (LS6500, Beckman Counter, USA).

Peptides were synthesized *via* conventional solid phase peptide synthesis (SPPS) by ChinaPeptides Co., Ltd. (Shanghai, China). HYNIC conjugated peptides were synthesized with developed method described before^40, 41^. Ethylene cysteine (EC) was first prepared and characterized using a reported procedure^42, 43^, and conjugated to peptides by Fmoc Solid-Phase Peptide Synthesis^44^.All the peptides were purified by HPLC and the purity of each peptide was beyond 95%.The mass spectrometry (MS) was showed in Figure S1.

The human lung adenocarcinoma cell line A549 was purchased from the Type Culture Collection of the Chinese Academy of Sciences (Shanghai, China) and maintained in high-glucose Dulbecco’s modified Eagle’s medium (DMEM) (HyClone, USA) supplemented with 10% fetal bovine serum (Gibco, USA) with 1% penicillin/streptomycin at 37 °C in a humidified atmosphere containing 5% CO_2_.

BALB/c-nude mice (5 weeks old) were purchased from the Experimental Animal Center of the Third Military Medical University (Chongqing, China) and were housed under specific pathogen-free (SPF) conditions.

Animal experiments, including xenograft model, SPECT planar imaging, biodistribution analysis and therapeutic evaluation, were performed according to protocols approved by the Laboratory Animal Welfare and Ethics Committee of the Third Military Medical University (Chongqing, China). The methods were carried out in accordance with the approved guidelines.

#### Peptide screening process

The procedure for peptide screening is shown in Fig. 1A. Briefly, the VEGFR extracellular immunoglobulin-like modules Ig1–Ig3 were used as targets to systematically alter the primary peptide sequence of VEGF_125-136_. Bioinformatics software (Autodock-Vina 1.1, Cn3D, VMD1.8.3) was used to simulate the interaction between VEGF_125-136_ and VEGFR-1 (Fig. 1B). The core motif of VEGF_125-136_, RKRKKSR, remained unaltered *in silico*; however, the amino acids at the 1st, 2nd, 10th, 11th, and 12th positions were sequentially mutated. Molecular docking and surface functional group interaction assays were performed to screen for optimized peptides.

Selected peptides were synthesized, and the 50% inhibitory concentration (IC_50_) values were determined based on receptor competition binding assays with ^125^I-VEGF_165_^28^.

The ^3^H-thymidine incorporation assay was performed to assess the inhibitory rate of peptides. First, A549 cells (6,000 counts per cell) were seeded in a 96-well plate and cultured overnight. The peptides were added at a final concentration of 240 μmol/L and incubated for 18 h. ^3^H-TdR was then added to each well at a radioactivity of 1 μCi and incubated for 6 h. After supernatant was removed and washed with phosphate-buffered saline (PBS) twice, the cells were digested and collected. The radioactivity was measured *via* liquid scintillation counting. The inhibitory rates were calculated by comparing the cell growth rate relative to the control groups.

#### ^99m^Tc/^188^Re-labeling and *in vitro* stabilities

Hydrazino-nicotinamide (HYNIC) or ethylene cysteine (EC) was conjugated to the peptide sequence as the chelating agent for ^99m^Tc or ^188^Re radiolabeling, respectively (Fig. 1D). The process for ^99m^Tc labeling was performed as previously described^45^. In brief, 50 mg Tricine, 5 mg TPPTS (as a reducing agent for Na[^99m^TcO_4_]) and 20 μg HYNIC-conjugated peptide were added to 0.25 mM succinic acid buffer (100 μL, pH = 5); then, 100 μL Na^99m^TcO_4_ (5 mCi) was added to the above-mentioned mixture; the reactions were performed at 100 °C for 20 min under nitrogen-purged conditions.

For ^188^Re radiolabeling, the following substances were added and mixed: 40 μL of EC-conjugated peptide (1 g/L), 50 μL of ammonium acetate buffer (pH 5.2), 40 μL of stannous chloride (10 g/L, containing 1 μg/μL ascorbic acid, prepared immediately prior to use), and 100 μL of tartrate buffer (50 g/L Na·K·tartrate·2H_2_O, 0.5 mol/L NaHCO_3_, 0.25 mol/L NH_4_Ac, and 0.175 mol/L NH_3_ ·H_2_O). Na^188^ReO_4_ (100 μL, 185 MBq) was added to the above-mentioned mixture. Then, the mixture was homogenized and sealed, followed by incubation under nitrogen-purged conditions at 37 °C for 30 min. Finally, to maintain the stability of ^188^Re-labeled peptide, ammonium perrhenate was added as a stabilizer. Following the same procedure, ^99m^Tc/^188^Re-labeled VEGF_125-136_ was prepared^27^.

The radiochemical purity (RCP) was determined using paper chromatography as follows. Whatman 3 MM paper served as the stationary phase. Free ^99m^TcO_4_^−^/^188^ReO_4_^−^ was detected (Rf = 0.9–1.0) using acetone as the mobile phase, and ^99m^Tc/^188^Re-colloid was measured (Rf = 0) using ammonia-ethanol-water (1:2:5) as the mobile phase. The radioactivity of the paper was measured using a γ counter. Labeling efficiency or RCP (%) = 100% − (free^99m^TcO_4_^−^/^188^ReO_4_^−^)% − (^99m^Tc/^188^Re-colloid)%. High-performance liquid chromatography (HPLC) was performed to purify and identify the labeled peptides.

HPLC experiments were performed using an e2695 Separation Module (Waters, USA) and a HYPERSIL C18 column (4.6 × 250 mm, particle size 5 µm; Waters, USA) to determine whether the peptide was successfully labeled by radionuclide. Briefly, the Sep-Pak C18 column was eluted with buffer A (0.1% trifluoroacetic acid in water) and a gradient (0–80%) of 0.1% buffer B (0.1% trifluoroacetic acid in acetonitrile) for 22 min at a flow rate of 1 mL/min with an absorbance wavelength of 220 nm. The eluates were collected with 10 drops per tube synchronously. The radioactivity of each tube was detected with a γ counter, and the time-radioactivity (T-A) curve was plotted.

The stabilities of the peptides were evaluated by the incubation of ^99m^Tc/^188^Re-labeled peptides with saline or human plasma at 37 °C for different periods of time (1, 2 or 6 h for ^99m^Tc-labeled peptide or 2, 6 or 24 h for ^188^Re-labeled peptide); then, RCP was calculated as described above.

#### Competitive receptor binding assays

Competitive receptor binding assays were performed as previously described with minor modifications^46^. Briefly, A549 cells (2 × 10^4^ counts/well) were seeded into a 24-well culture plate and cultured overnight. Gradient concentrations (0, 0.6, 1.2, 6, 30, and 60 nmol/mL) of the non-chelated and non-labeled peptides were added and incubated 30 min prior to corresponding ^99m^Tc/^188^Re-labeled peptide (0.1 nmol/mL, 370 KBq) addition. Two hours later, cells were washed three times with ice-cold PBS containing 0.1% BSA and collected. The radioactivity was measured with a γ counter. Furthermore, to determine whether the chelating agent could affect the activity of peptides, HYNIC-peptide and EC-peptide were tested. The IC_50_ values were calculated by fitting the data to a nonlinear regression using Graph-Pad Prism 5.0 (GraphPad Software, Inc.).

#### Construction of the VEGFR-1-overexpression model of lung cancer

VEGFR-1-overexpression A549 cells were used to verify the binding abilities of the peptides to VEGFR-1. In brief, a mutation was inserted into the ATP-binding site of the VEGFR-1 gene to avoid additional influence on the tumor cells. A VEGFR-1 overexpression lentiviral vector (Lenti-VEGFR-1) and a mock vehicle were constructed by NeuronBiotech Co. (Shanghai, China). A549 cells (5 × 10^4^ counts/well) were seeded in 6-well plates overnight, and the cells were transfected by lentiviral vector at a multiplicity of infection (MOI) of 50 using 5 μg/mL polybrene (Sigma, USA). After incubation for 24 h, the medium was replaced with fresh virus-free medium. After the cells were passaged 3 times, real-time polymerase chain reaction (RT-PCR), immunofluorescence staining and western blot (WB) analysis were performed to evaluate VEGFR-1 expression.

#### Saturation binding and internalization assays

A549 cells transfected with Lenti-VEGFR-1 or the mock vehicle (2 × 10^4^ counts/well) were seeded in a 24-well plate and incubated overnight. For the saturation binding assay, 30 μg of the non-labeled peptides was added 30 min prior to gradient concentrations of ^188^Re-labeled peptide incubation. Medium was removed 2 h later, and cells were washed three times with ice-cold PBS containing 0.1% BSA and collected; the radioactivity of the cells was detected using a γ counter.

The internalization assay was performed as previously described^47, 48^ with minor modifications. Briefly, the cells were incubated with ^188^Re-labeled peptide (7.4 MBq, 0.1 μg of peptide). For blocking assay, 1,000-fold molar excess of the non-labeled peptides was added simultaneously with ^188^Re-labeled peptide. Internalization was terminated at different incubation times (15 min to 4 h), and cells were washed twice with ice-cold PBS containing 0.5% BSA to remove non-specific binding peptide. ^188^Re-labeled peptide specifically bound to the cell surface was collected by washing the cells twice with 20 mmol/L sodium acetate (pH 2.8, ice cold) for 5 min. Then, the cells were collected for radioactivity detection of internalization peptide. The percentage of intracellular radioactivity relative to the total radioactivity at each time point was calculated as the internalization rate.

#### Co-immunoprecipitation to identify the binding between peptide and VEGFR-1

GST-peptide was prepared and purified as previously described. Briefly, pGEX4T-1-peptide plasmid was obtained by peptide gene insertion into pGEX4T-1. Then, the plasmids were transfected into *E*. *coli* BL21 (DE3)-competent cells. Subsequently, GST-peptide fusion proteins were collected and purified by Glutathione Sepharose 4B. The obtained proteins were identified by protein electrophoresis.

A549 cells were seeded in 75 cm^2^ bottles (1 × 10^4^ counts/mL). After 24 h, GST-QKRKRKKSRKKH (80 nmol/L) or GST–RKRKRKKSRYIVLS (185 nmol/L) protein was added, respectively. After 12 h, the culture medium was removed and the cells were washed by PBS and then spalled with IP lysis buffer containing protease inhibitor (4 °C, 30 min). The protein concentration was determined with BCA methods. Protein (600 μg) from supernatant was used for immunoprecipitation (IP) assays with 1 μg anti-GST antibody or 1 μg anti-VEGFR-1 antibody in a reversed Co-IP experiment, while IgG served as the negative group. After washing 3× by TBST, IgG-HRP was added, shaking 2 h at RT, and last for enhanced chemiluminescence imaging.

#### Preparation of xenograft tumor animal models

Five-week-old male BALB/c-nude mice were used for preparation of the xenograft tumor models. An A549 lung carcinoma-bearing model was established by subcutaneous injection of A549 cells at a concentration of 5 × 10^6^ cells/mouse into the right front flank of the mice.

#### SPECT planar imaging

SPECT planar imaging was performed when the tumor volume was approximately 1 cm^3^. The mice were anesthetized and placed in a supine position. Planar imaging was performed using a low-energy collimator at an energy peak of 140 keV at the indicated time after ^99m^Tc-labeled peptide (11.1 MBq, 150 μL) injection. The main imaging acquisition parameters were as follows: 128 × 128 matrix, window-width 20%, zoom 2.0, acquisition time 10 min. For ^188^Re imaging, a high-energy collimator at an energy peak of 155 keV was used. Then, 100,000 counts were acquired per image at each time indicated after the injection. For the blocking study, an excessive amount of HYNIC-peptide or EC-peptide (500 μg/mouse) was injected 30 min prior to radiolabeled peptide injection.

#### Biodistribution analysis of the ^188^Re-labeled peptides

Twenty-two A549 tumor-bearing nude mice were randomly divided into 2 groups (n = 12) and *i*.*v*. injected with ^188^Re-labeled peptide (7.4 MBq, 150 μL). The mice were euthanized at various time points (2, 6 h) after injection. Then, samples of blood, muscle, tumors and main organs (heart, liver, spleen, lung, kidney, brain) were collected, weighed and measured using a γ counter. The percentage of injected dose per gram (% ID/g) was calculated using a correction for background radiation and physical decay.

#### Therapeutic evaluation of ^188^Re-peptides on A549 tumors

Sixty mice were randomly separated into 6 groups once the tumor volumes reached approximately 0.1 cm^3^: saline group, ^188^ReO_4_^−^ group (18.5 MBq), non-labeled peptide group (4 μg per mouse) and ^188^Re-labeled peptide group (18.5 MBq, 4 μg peptide per mouse) (n = 10). All drugs were administered *i*.*v* at the first and seventh day. Tumor size was determined weekly by measuring the length, width, and depth of the tumor using a caliper. Tumor volume was calculated as follows: tumor volume = (length × width × depth) × π/6. The mice were abandoned once dead or body weight loss was more than 20%.

At the end of the therapeutic period, mice were sacrificed, and the tumors were collected and fixed in 4% paraformaldehyde (PFA). Routine hematoxylin and eosin (H&E) staining and TdT-mediated dUTP nick-end labeling (TUNEL) staining were performed for necrosis and apoptosis analysis according to the manufacturer’s instructions.

#### Statistical analysis

Experimental data were represented as the mean ± standard deviation (SD). Statistical analysis was carried out *via* Student’s unpaired *t* test between two groups or single-factor analysis of variance (ANOVA) with a multiple comparison method for tests consisting of more than 2 groups. *P* < 0.05 was considered significant.

## Electronic supplementary material

Supplement information
