# Supplementary material for: Novel small peptides derived from VEGF125-136: potential drugs for radioactive diagnosis and therapy in A549 tumor-bearing nude mice
Source: Sci Rep. 2017 Jun 27;7:4278. doi: 10.1038/s41598-017-04513-y (PMC5487319; doi:10.1038/s41598-017-04513-y)
Supplement: Supplementary file 1 — Supplement information [file 41598_2017_4513_MOESM1_ESM.doc]

**Novel small peptides derived from VEGF_125-136_: potential drugs for radioactive diagnosis and therapy in A549 tumor-bearing nude mice**

Xiang Zhang^#1^, Shibin Feng^#1^, Jie Liu^1^, Qianwei Li^1^, Lei Zheng^1^, Laiping Xie, Hongmin Li^1^, Dingde Huang*^1^

^1^ Department of Nuclear Medicine, Southwest Hospital, Third Military Medical University, 30 Gaotanyan Street, Shapingba District, Chongqing 400038, China. ^#^ These authors contributed equally to this work. Correspondence and requests for materials should be addressed to D.H. (email: huangdde@tmmu.edu.cn).

**Methods**

***HPLC Analysis of Peptide Adducts***

Prior to HPLC analysis, tubes containing peptides were centrifuged at 14000g for 5 min and the supernatant was loaded into HPLC analysis vials. HPLC separation of peptides: an HYPERSIL C18 5um column; mobile phases: A, acetonitrile:water: trifluoroacetic acid =5：94.85：0.15 and B, acetonitrile:water: trifluoroacetic acid =90：9.9：0.1; Flowrate: 1.0 ml/min; Pressure: 10.0 MPa; Length: 250 mm ;Detector: UV 220 nm; Diameter:4.6 mm; Sample Volume: 80 ul. The elution were collected and the radioactivity was measured by γ-counter.

***VEGFR-1 Gene Expression Analysis by Quantitative Reverse Transcriptase Polymerase Chain Reaction (Q-RTPCR)***

Total RNA of A549 cells was extracted using standard TRIZOL (Invitrogen) method. cDNA was synthesized using First Strand cDNA Synthesis Kit (TOYOBO) according to the manufacturer’s instructions. Real-time PCR analysis was performed using 2.5 μL of cDNA and 10 pmol of gene specific primers (Table S4). The comparative threshold cycle (Ct) values were calculated using CFX 96 Real-Time PCR system (Bio-Rad Laboratories, CA, USA) using SYBR Green chemistry (Bio-Rad).

***Western blotting***

Cellular protein was isolated by RIPA buffer (50 mM Tris-HCl, 5 mM EDTA, 150 mM NaCl, 1% NP-40 (v/v), 0.1% SDS (w/v), 0.5% sodium deoxycholate (w/v), pH 8.0) containing complete protease inhibitors (Invitrogen). Protein samples were mixed with loading buffer, heated at 100 °C for 5 min, separated using 12% SDS-polyacrylamide gel electrophoresis, and transferred onto polyvinylidene fluoride membranes (Merck Millipore, Germany). VEGFR-1 was immunolabeled with rabbit anti-human VEGFR-1 monoclonal antibody (Santa, USA) at 4 °C overnight. Membranes were washed and then incubated with horseradish peroxidase-conjugated (HRP) secondary antibodies for 1 h at room temperature. The protein bands of VEGFR-1 were exposed by Gel-Doc 2000 (Bio-rad,USA) using ECL Plus Detection Reagent (Merck Millipore, Germany). β-actin was used as an internal control.

**Results**

**
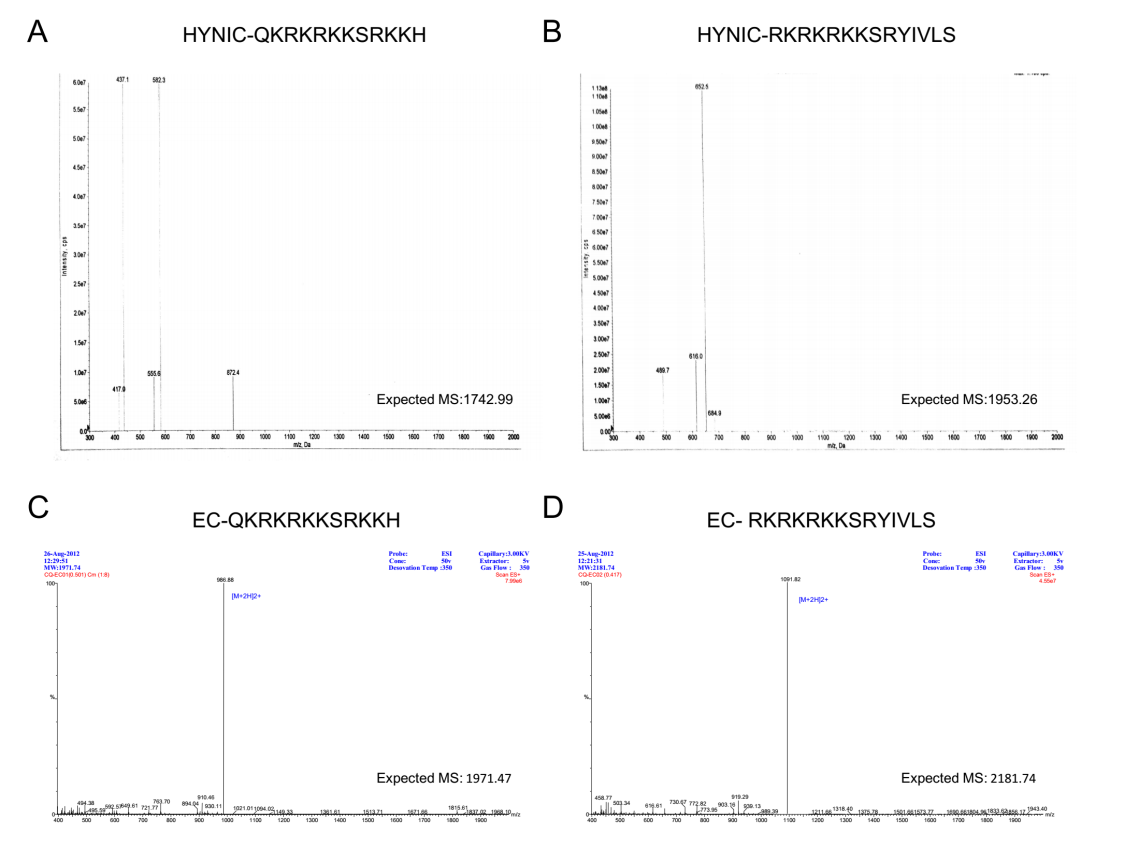
**

**Figure S1** Mass Spectrometry (MS) of HYNIC conjugated peptides (**A,B**) and EC conjugated peptides (**C,D**).


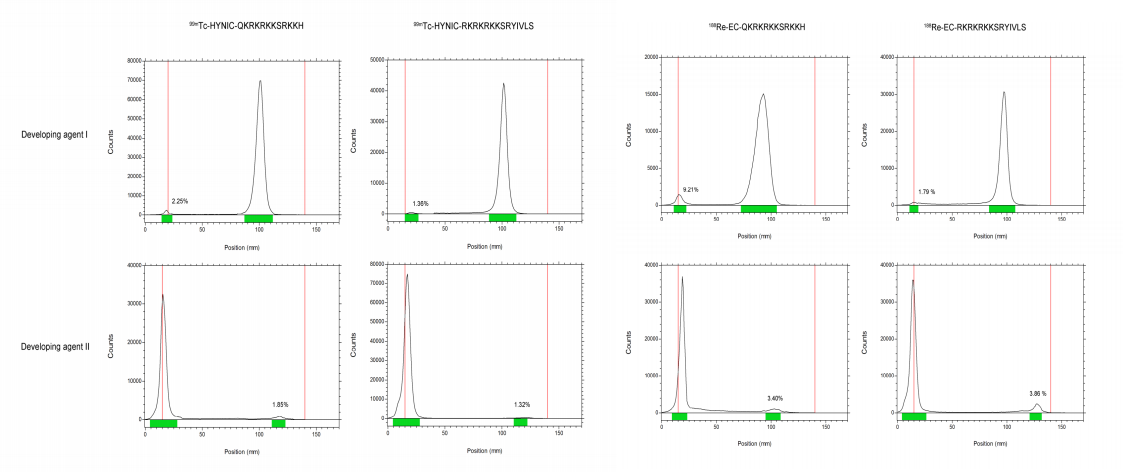


**Figure S2** Paper chromatography of ^99m^Tc-peptides and ^188^Re-EC peptides. Developing agent I :ammonia-ethanol-water (1:2:5); Developing agent II: acetone.

**
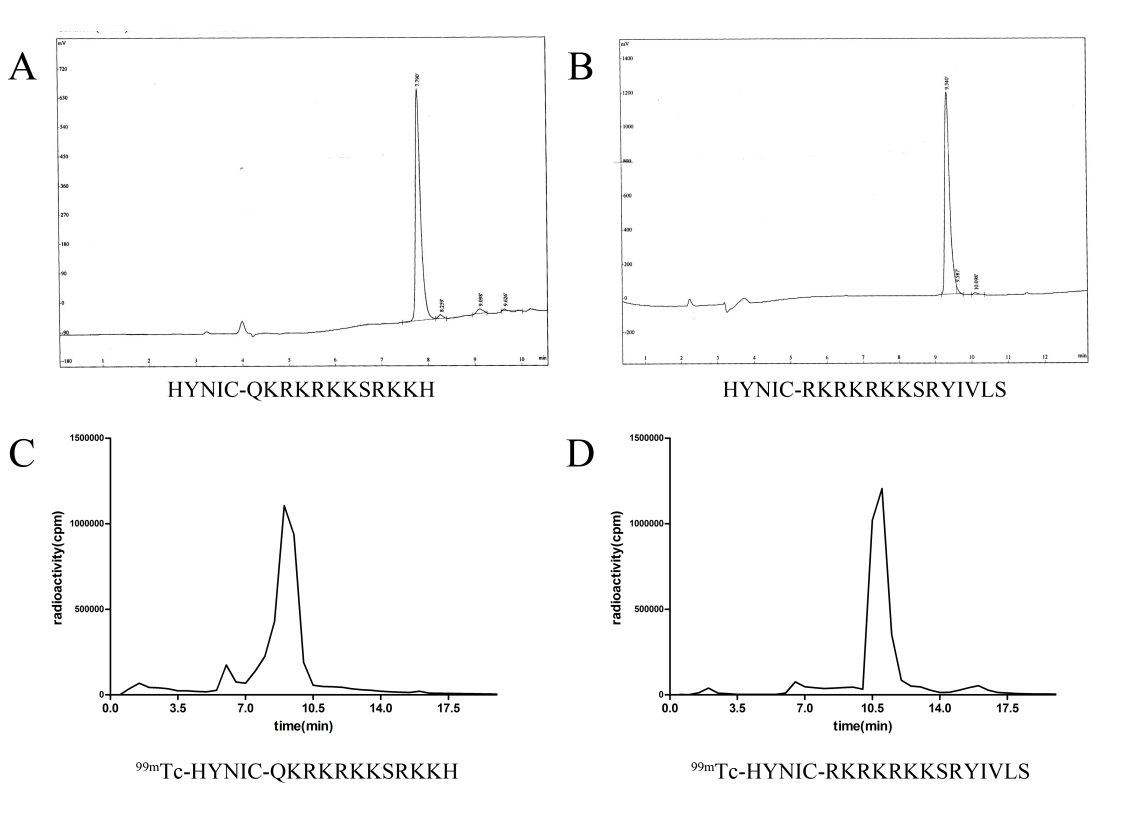
**

**Figure S3** HPLC analysis of HYNIC conjugated peptides (**A,B**)and time-activity cure of ^99m^Tc-HYNIC peptides(**C,D**).

**
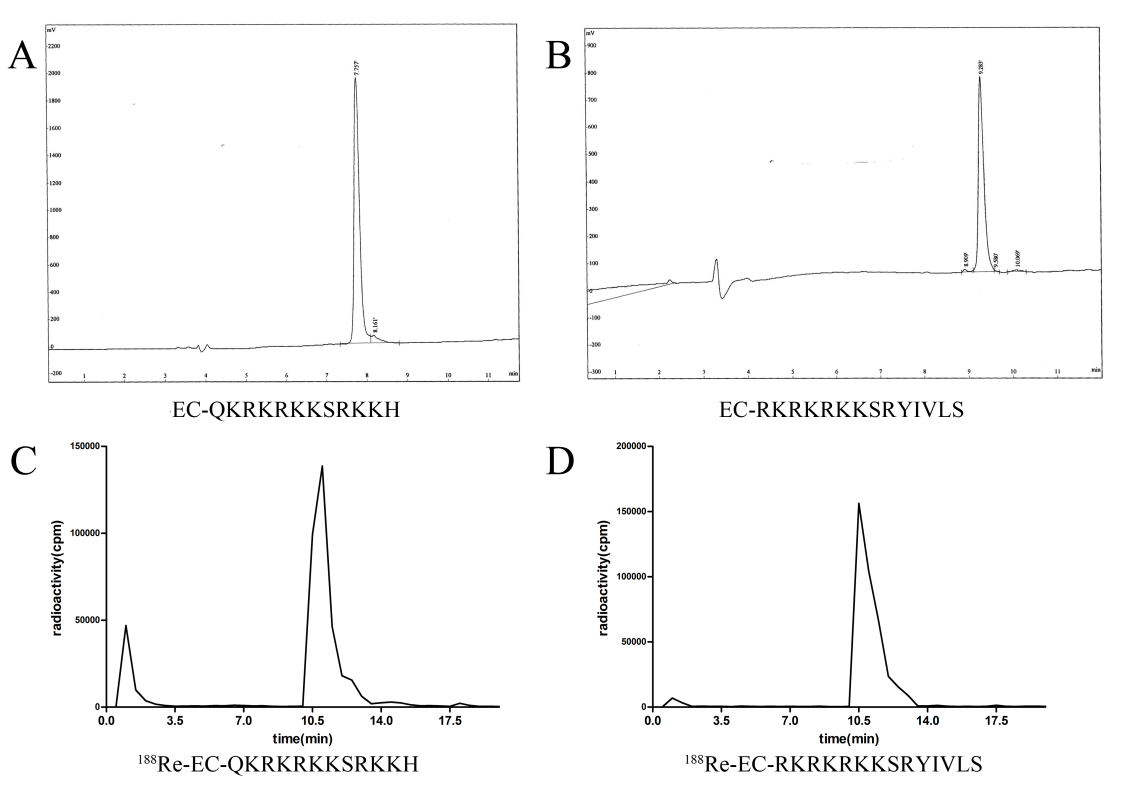
**

**Figure S4** HPLC analysis of EC conjugated peptides (A,B)and time-activity cure of ^188^Re-EC peptides(C,D).


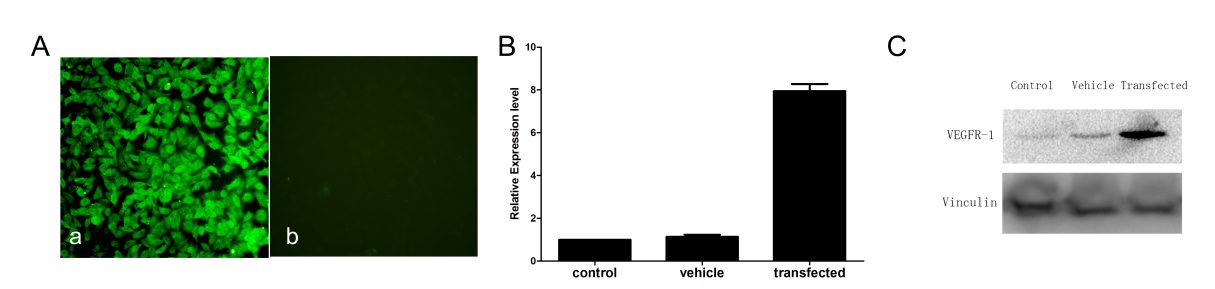


**FIGURE S5. (A)** Indirect immunofluorescence for the Flag-tagged protein encoded by the lentiviral vector was stronger in the transfected A549 cells (a) compared with the control cells (b). (**B)** Relative expression of VEGFR-1 mRNA in A549 cells was detected via real time RT-PCR analysis. **(C)** The expression of VEGFR-1 in A549 cells evaluated via western blot analysis. Vinculin was used as an internal control.


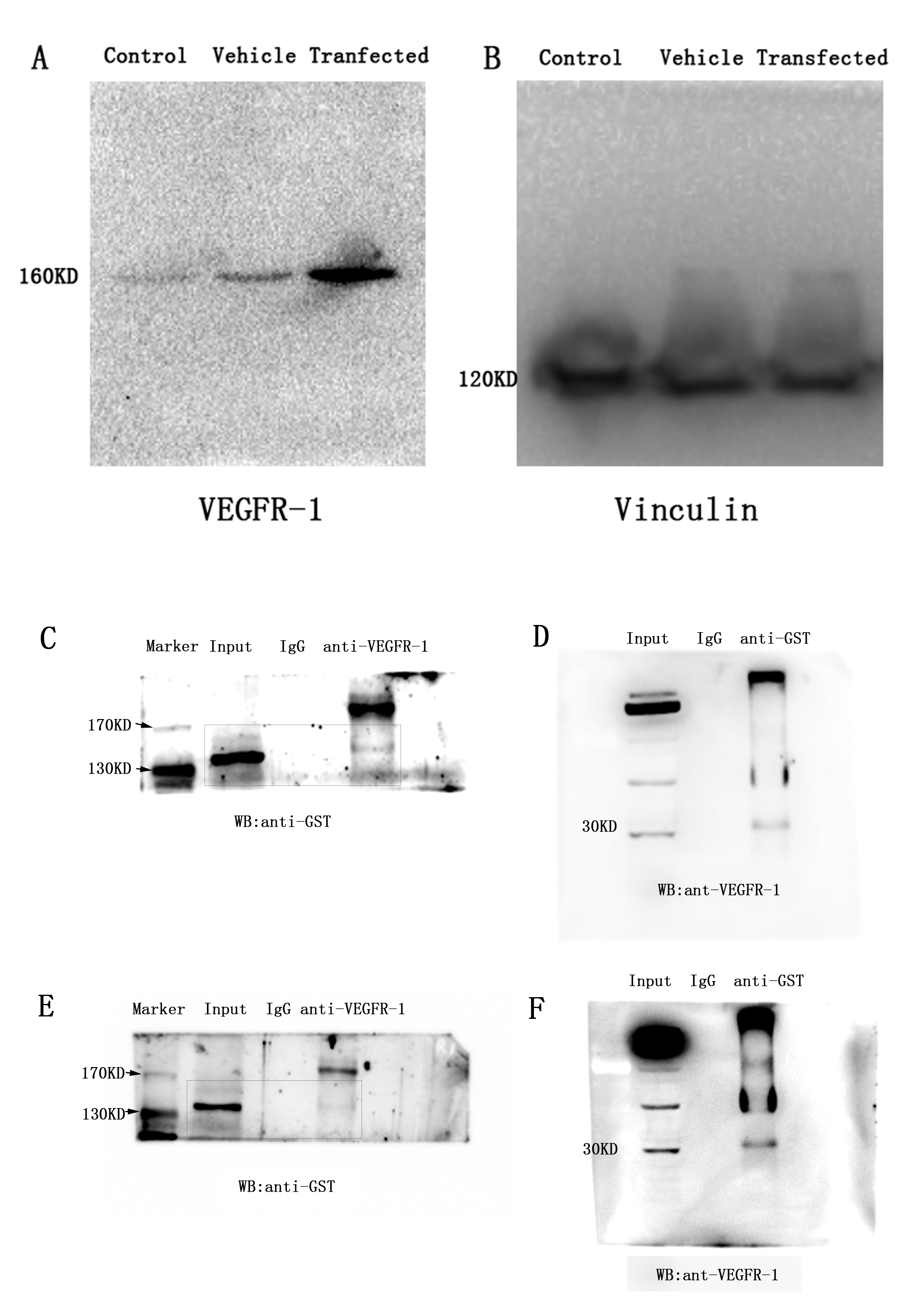


**FIGURE S6. (A,B)** Full length blots of the expression of VEGFR-1 in A549 cells. Corresponding to FIGURE S3, C. **(C,D)** Full length blots of co-immunoprecipitation of GST-QKRKRKKSRKKH and VEGFR1. **(E,F)** Full length blots of co-immunoprecipitation of GST-RKRKRKKSRYIVLS and VEGFR1.Corresponding to Figure 4 E,F.


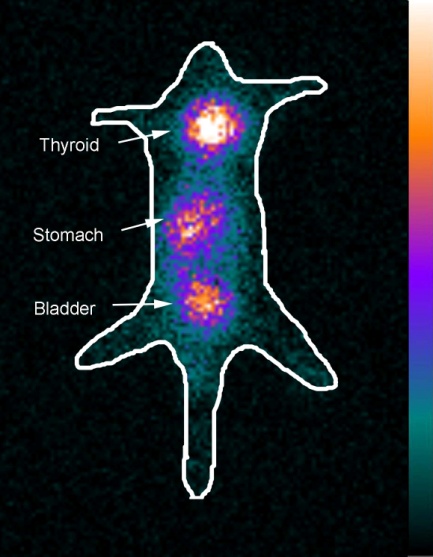


**Figure S7.** SPECT planar imaging of A549 tumor-bearing nude mice after intravenous administration of ^188^Re-perrhenate at 2 hour. Large amount of radioactivity was observed in thyroids and stomach.

**Table S1.** Sequences of primers for Real-Time PCR.

| *Names* | **Sequences** |
| --- | --- |
| **VEGFR-1** | Sense primer 5’-GAAGGCATGAGGATGAGAGC-3’  Antisense 5’-CAGGCTCATGAACTTGAAAGC-3’ |
| **β-actin** | Sense primer 5’-ACCCCGTGCTGCTGACCGAG-3’  Antisense 5’-TCCCGGCCAGCCAGGTCCA-3’ |
